# Addressing energy insecurity: Policy Considerations for enhancing energy assistance programs

**DOI:** 10.1016/j.heliyon.2024.e24178

**Published:** 2024-01-23

**Authors:** Michelle Graff

**Affiliations:** Maxine Goodman Levin School of Urban Affairs, Cleveland State University, 2121 Euclid Avenue, Cleveland, OH, 44115, USA

**Keywords:** Energy assistance, Energy insecurity, Energy justice, Public assistance

## Abstract

Household energy consumption is crucial for a productive and safe life. Despite its importance, 33.7 million U.S. households experienced energy insecurity in 2020. This paper examines the prevalence, correlates, and effects of energy bill assistance programs, which aim to alleviate the hardship. This analysis relies on logistic regressions and the 2020 Residential Energy Consumption Survey, a nationally representative survey administered by the Energy Information Administration. Results reveal 16 percent of the energy insecure population received assistance to pay its energy bill in 2020. Several socioeconomic and housing characteristics are associated with receipt of assistance; however, logistic regression estimates suggest prior participation in energy assistance and receipt of a disconnection notice from a utility provider are indicators substantively and significantly associated with energy assistance participation that warrant attention from scholars and practitioners. Lastly, outcomes generally indicate previous participation in energy assistance does not impact the odds a household will experience energy insecurity. Based on findings, I offer three policy recommendations: 1) increase resources spent on energy assistance to align with demand; 2) enhance communication between utility providers and low-income households regarding available assistance opportunities; and 3) prioritize engagement with populations that never participated in energy assistance to facilitate successful application processes.

## 1 Introduction

Household energy consumption enables the provision of essential residential services, such as lighting, heating/cooling, refrigeration, cooking, and the use of electronic and medical devices. Furthermore, energy plays a vital role in facilitating connections to social networks, colleagues, and educational services. In the U.S., 33.7 million households endured financial hardships resulting from non-discretionary energy consumption, like cooking [1,2], despite reducing energy use, bill balancing, accumulating debt, and sacrificing necessities like food and medicine to pay energy bills [[3], [4], [5]].

Energy insecurity – inability to meet household energy consumption needs [6] – disproportionately impacts vulnerable populations [see e.g., [7]] and is a social problem of increasing concern to public health, policy, and social scientists. Losing access to power cuts individuals off from life's necessities, and studies document limiting household energy consumption is associated with poor health outcomes, including mortality [[8], [9], [10], [11], [12]].

Given the upward trend in energy costs due to fluctuating global wholesale prices [13] as well as the impacts of (e.g., heat waves and erratic weather) and responses to (e.g., investments in decarbonization infrastructure and technology) climate change, energy bills will likely continue to climb as utilities seek rate increases to cover expenses [see [14]]. Consequently, more households will require financial assistance to meet monthly energy demands [15]. In fact, recent findings suggest low-income households, even if they have adopted energy-efficient technologies, will require assistance to pay their energy bills to fulfill their essential energy needs [16]. Historically, however, programs that help households maintain access to power, such as the federal block-grant Low Income Home Energy Assistance Program (LIHEAP), only help 16 to 22% of the income-eligible population each year [17].

Scholarly attention to energy insecurity and related hardships, such as energy burden (proportion of income spent on energy), is increasing [18]. However, fewer studies have investigated the energy assistance programs designed specifically to help households pay their energy bills. In this paper, I aim to provide insights into U.S. energy bill assistance by exploring the prevalence, determinants, and impacts of participation in these programs. I use the 2020 Residential Energy Consumption Survey (RECS) – a nationally representative survey administered by the Energy Information Administration (EIA) – to conduct the analysis.

Results from logistic regressions confirm participation in energy bill assistance programs is low relative to demand. According to the RECS, 16% of energy insecure households received assistance in 2020. Empirical models identify determinants of energy assistance recipients, finding several socioeconomic, housing, and energy insecurity measures are significant and substantive indicators. Notably, previous participation in energy assistance and receipt of a notice from a utility provider threatening to disconnect a household's power are associated with the highest odds of receiving assistance in 2020, indicating these measures may have the most substantial impact on likelihood of energy assistance participation. These results uncover key pathways – prior assistance, formal communication from a utility provider, and a household's urgent need for financial support – associated with participation in energy assistance. Lastly, results indicate previous participation in energy assistance programs is not linked with a reduction in a household's likelihood to experience energy insecure conditions, underscoring the insufficiency of current programs in addressing the financial burdens imposed by energy bills on households.

Therefore, I conclude with three policy recommendations. First, decisionmakers must increase support for energy bill assistance programs to align with demand. Second, utility providers should connect with low-income households more frequently, especially those with missed or partial payments, to provide information about relevant energy bill assistance opportunities. Lastly, administrators should prioritize engaging with households that have yet to participate in assistance, guiding them through the application process, because previous participation has the largest estimated correlation with future program participation.

## 2 Literature review

Beginning in the late 1980s, scholars raised concerns about measuring poverty with income-based metrics [[19], [20], [21], [22]] because income alone is a unidimensional assessment, whereas poverty is a multidimensional problem [23]. For this reason, researchers focused on material hardships, which refer to situations where households cannot meet their basic needs [[24], [25], [26]]. Material hardship research largely explores a household's (in)ability to pay their rent or mortgage or afford nutritious food [see e.g., [[27], [28], [29]]]. Hernández and Laird (2022) [30] argue the expansive food and housing insecurity research laid the foundation for conceptualizing appropriate policy remedies, like food stamps and housing subsidies.

In contrast, energy insecurity and other related financial hardships (e.g., transportation, water, childcare) are often conflated into a single essential expenses category [see e.g., [31]]. Heflin et al. (2009) [32] cautions against lumping together diverse dimensions of material hardship because the results may be misleading, potentially resulting in inappropriate conceptual frameworks and impractical policy solutions. Bednar and Reames (2020) [33] further argue lack of formal acknowledgment of energy insecurity as a distinct material hardship has limited the U.S. federal government's response to the widespread problem.

Consequently, household energy insecurity and its related public assistance programs remain comparatively less understood despite the importance of consistent residential energy consumption to financial stability [34], physical health [11,35,36], and mental wellbeing [37,38], especially for children [39] and the elderly [40,41].

### 2.1 Energy insecurity literature

Data reveals American households struggle to maintain consistent access to energy due to financial obstacles. In 2017, 25.8 million – 67% – of low-income households faced high energy burdens (i.e., paid more than 6% of income on energy bills) [42], with vulnerable characteristics – historically marginalized communities and tenants – often accompanying higher energy burdens [43]. In 2020, over a quarter of the U.S. population could not pay an energy bill or reduced their energy consumption to unsafe or uncomfortable indoor temperatures [2].

Multiple terms capture the inability to consume or afford adequate energy [see e.g., [44]], such as fuel poverty [[45], [46], [47]], energy poverty [48], and energy justice [49,50]. Hernández (2016) [6] specifically conceptualized residential energy insecurity using three interrelated dimensions: 1) economic – financial challenges households encounter while covering monthly energy expenses; 2) physical – deficiencies in the infrastructure of a home; and 3) behavioral – coping strategies employed to alleviate the effects of economic and physical energy insecurity. In the last decade, scholarship focused on the economic and physical elements of energy insecurity, providing foundational insights.

National [see e.g., [[51], [52]]], regional [53], and local [50,54] research reveals vulnerable populations disproportionately experience both economic and physical energy insecure conditions. Using the American Community Survey, Lyubich (2020) [55] discovers Black households have higher energy expenditures than white households. Drehobl et al. (2020) [42], based on the American Housing Survey, finds Black households along with low-income, Hispanic, and renters have disproportionately higher energy burdens than the national median household. Moreover, Konisky and colleagues (2022) [56] as well as Memmott et al. (2021) [7] survey low-income U.S. households and find Black, Hispanic, and medically compromised individuals are less likely to be able to pay monthly energy bills and more likely to receive disconnection notices and be disconnected from their utility provider. State-level data in Illinois additionally finds Black and Hispanic zip codes are 4–5 times more likely to be disconnected from their utility provider for nonpayment [57].

Relying on previous iterations of the RECS, Hernández and Laird (2022) [30] and Best and Sinha (2021) [58] found it is not only income, respondent characteristics, or housing tenure that is associated with economic energy insecurity. In fact, these studies and Graff and colleagues (2021) [59] find those reporting physical energy insecurity (e.g., poor insulation) were more likely to experience economic energy insecurity as well. Similar research finds disparities across income and race when examining access to and adoption of energy efficient and electric technologies, which not only help meet climate change mitigation goals, but they also improve the infrastructure of a home (i.e., mitigate physical energy insecurity), and reduce energy costs (i.e., alleviate economic energy insecurity) [see e.g., [[60], [61]]]. Studies, importantly, connect disparities in physical and economic energy insecurity with historical discriminatory housing policies, such as redlining and exclusionary zoning [62,63].

Extant literature also provides insights into behavioral energy insecurity. When low-income U.S. households struggle to pay their energy bills, over 50% engage in at least one behavioral coping mechanism [4]. These mechanisms range from wearing extra layers and using blankets [64] to only heating specific rooms in their homes [65], using less electricity or fuel, turning on heat or air conditioning once indoor temperatures became uncomfortable or unsafe [[5], [66]], and utilizing unsafe devices (e.g., propane heaters) which exacerbate health risks [67]. Other analyses document financial strategies households employ to pay energy bills, including accruing debt; bill-balancing; taking out high-interest loans; receiving help from friends, family, or local organizations; signing up for utility payment plans; or cutting expenditures, such as food or medicine [[3], [34], [57], [68], [69], [70]].

Notably, literature consistently reveals a limited number of income-eligible households “seek government assistance to cope with energy insecurity, despite this being one of the least risky strategies” [[4], page 3]. U.S. energy bill assistance programs, including LIHEAP, help households pay for either heating or cooling; assist households during a crisis (e.g., receipt of disconnection notice, disconnection, broken equipment); or support weatherization projects (e.g., energy efficient windows) [71]. The average size of an annual LIHEAP household heating (cooling) benefit was $429 ($439) in 2020, and once financial support for an energy bill is offered to those that qualify, payment is often sent directly to utility providers [72]. Hernández and Laird (2022) [30], however, find those disconnected or at risk of being disconnected from their utility provider most often forgo necessities to pay an energy bill, with less than 10% seeking publicly-funded energy assistance.

Limited participation in government-sponsored energy bill assistance is likely a function of multiple factors. First, interviews with low-income Boston residents reveal resources supporting energy assistance programs are quickly exhausted, especially in urban areas [6]. Between 2017 and 2021, Congress appropriated LIHEAP between $3 and $4 billion each year, providing energy bill support to approximately 5–6 million households – 15 to 17% of the income-eligible population [72]. In comparison, federal food stamps – Supplemental Nutrition Assistance Program (SNAP) – was allocated $60.4 billion and served approximately 35 million people, approximately 80% of the eligible population, in 2019 [73], highlighting a disparity in federal funding prioritization between food and energy insecurity.

Second, Treadway (2018) [74] found many individuals were either unaware of energy assistance opportunities or confused about how to apply. Household eligibility for state- and federal-sponsored energy assistance varies across states and providers as programs are often administered through local-level providers, such as Community Action Agencies (CAA). Like other public assistance programs, eligibility is based on income – often set at either 1) 150% of the federal poverty line (FPL) or 2) 60% of a state's median income (SMI) – and applicants must provide identification and income verification. However, energy assistance applicants face unique administrative burdens, or obstacles [see e.g., [75]], when applying for energy bill assistance that likely leads to confusion and limits participation [see e.g., [[71], [76]]]. For example, energy assistance applicants must submit additional documentation, such as utility provider and bill information. Additionally, because energy assistance is a household – not individual – benefit, tenants often need to get permission from landlords to apply for assistance and multifamily households must coordinate applications. Lastly, due to funding constraints, some energy assistance benefits, like LIHEAP, are only available during select months of the year.

## 3 Research questions and expectations

The existing literature highlights coping mechanisms households adopt to address economic and physical energy insecurity. However, less research focuses on the determinants and consequences of participating in one of the least risky coping strategies: targeted energy bill assistance. The objective of the present analysis is to provide insights into U.S. energy bill assistance programs that will inform effective responses to address household energy insecurity.

To do so, I ask two interrelated research questions. The first research question has two parts. First, it asks: what are the socioeconomic and housing characteristics associated with participation in energy bill assistance? Next, I query: is previous participation in energy bill assistance a significant predictor of future receipt, controlling for other characteristics?

Based on extant scholarship, I anticipate populations with vulnerable characteristics, including those with young children and individuals with medical conditions, as well as those experiencing energy insecurity are more likely to learn about payment assistance options [4]. Furthermore, I expect prior receipt of assistance will positively predict future assistance for two reasons. Firstly, Higgins and Lutzenhiser (1995) [77] found households participating in other public assistance programs (e.g., SNAP) had a higher likelihood of also participating in energy assistance, suggesting previous or ongoing engagement with social services is likely an indicator of future participation. Secondly, contrary to the assumption that households who previously received assistance would no longer require it in the future, Konisky et al. (2022) [56] discovered energy insecurity remains a persistent issue. This suggests households will continue to need support to meet their energy consumption needs and cover associated expenses; therefore, previous participation likely predicts future participation.

The second research question asks: is previous receipt of energy bill assistance correlated with energy insecure conditions in the future? If a correlation exists, does previous participation in energy bill assistance mitigate future energy insecure conditions?

As noted, while we might expect previous receipt of assistance to alleviate energy insecurity, scholarship on the topic is limited and mixed. Relying on the 2005 RECS, Murray and Mills (2014) [78] finds participation in energy assistance reduces a household's aggregated energy insecurity score; however, Memmott et al. (2021) [7] – through a longitudinal survey of low-income U.S. households between 2020 and 2021 – finds participation in any government assistance is associated with higher rates of being unable to pay an energy bill, receipt of a disconnection notice, and being disconnected from the grid. Consequently, I generate an aggregate energy insecurity measure and consider independent dimensions of energy insecure conditions in forthcoming analyses. Due to the mixed findings, I hypothesize receiving energy assistance may have minimal to no effect on household energy insecurity.

Furthermore, I investigate which households are more likely to experience energy insecurity without receiving bill assistance, thereby identifying particularly vulnerable populations. This contribution is significant for both researchers and practitioners, as it sheds light on those who are susceptible to energy insecurity but are not benefiting from bill assistance programs aimed at alleviating the hardship.

### 3.1 Summary

As I consider the literature on behavioral energy insecurity, a paradoxical relationship emerges. The paradox between the determinants of energy insecurity, including energy insecure conditions, and receipt of energy bill assistance poses uncertainties about the cyclical nature of the measures. These dynamics are likely to be different in each household; however, the purpose of the present inquiry is to empirically tease out, on average, the respondent level predictors associated with participation in energy assistance and subsequently to determine if receipt of assistance in a previous year affects energy insecure conditions in the future.

**Fig. 1:**
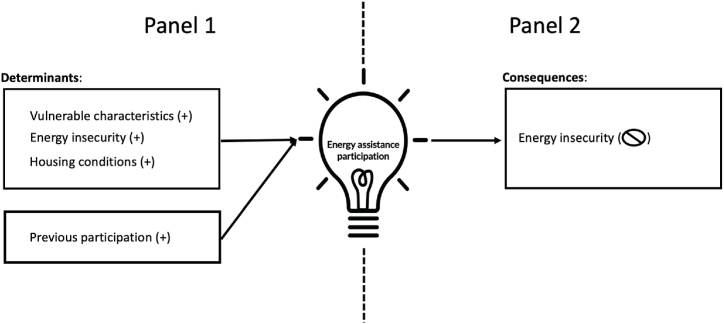
Framework for assessing determinants and consequences of residential energy assistance.

Fig. 1 illustrates the framework empirically investigated.

In Fig. 1, the left panel – 1– illustrates the relationships explored in the first research question: respondent-level determinants of energy bill assistance recipients. Specifically, I identify the expected positive relationship between receipt of energy bill assistance and vulnerable characteristics, energy insecure conditions, and deficient housing conditions. I additionally note previous participation in assistance is likely to be positively correlated with future receipt of assistance.

**Fig. 2:**
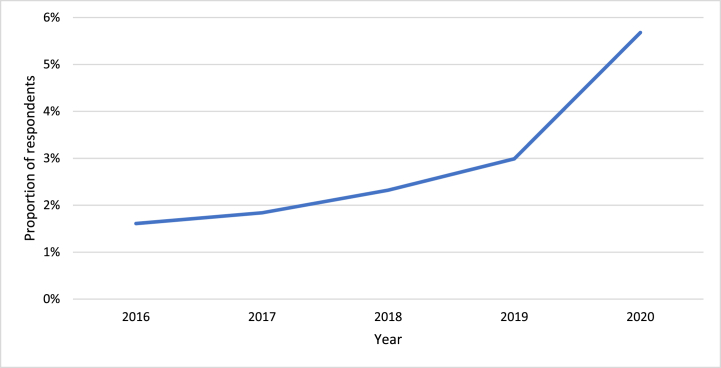
Weighted proportion of respondents receiving energy assistance, by year (Observations = 18,496; Population size = 123,529,025 occupied housing units) Note: Measures are weighted proportions, calculated using 60 jackknife replications.

The second part of the framework in Fig. 2 – panel 2 – examines the second research question. Specifically, it displays prior receipt of energy bill assistance is expected to yield a null impact on future household energy insecure measures. The two parts of the framework – panels 1 and 2 – jointly illustrate the circular, or bidirectional, relationship between residential energy insecure conditions and receipt of energy assistance. Therefore, disentangling causality between energy assistance and residential energy insecurity is a limitation (discussed in detail below). However, by observing this relationship, we can begin to assess the household characteristics associated with receipt of energy assistance and evaluate the feedback loop amongst receipt of assistance and energy insecure conditions.

## 4 Data and methods

The study analyzes the 2020 RECS, a nationally representative single-stage probability survey conducted by the EIA [2]. Survey data are cross-sectional, collected between September 2020 and April 2021. The EIA collected informed consent from all participants, as stated in the introductory questionnaire text [[80], page 4]. The 2020 RECS estimates energy and household characteristics for 18,496 respondents that statistically represent 123,529,025 million U.S. occupied housing units. 73% of respondents completed the survey by web and 27% by paper, with a weighted 37.9% response rate. The EIA employed hot-deck imputation to alleviate nonresponse bias and relied on the Jackknife method for variance and standard error estimation due to its single-stage stratified design [79].

I examine the 2020 RECS to answer my two research questions because it contains rich respondent and housing characteristics, provides information about energy consumption practices and energy insecurity experiences, and identifies if and when a respondent participated in energy bill assistance. While other articles have leveraged previous RECS to explore domestic energy insecurity and related programs [30,53,77], my analysis contributes to the scholarship because it leverages the 2020 RECS’ new state-level identifiers^1^ to empirically interrogate energy assistance recipients in relation to individual-level socioeconomic indicators, energy insecurity measures, and housing characteristics. To do so, all forthcoming models include state indicator variables, designated as state-level fixed effects. I do not limit the analysis to low-income households; instead, the analysis considers the full RECS sample because previous scholarship notes energy insecure conditions do not occur in only low-income households [5].

Table 1 displays descriptive statistics, including the weighted frequency and proportions, for all variables included in forthcoming analyses.

**Table 1:** Weighted descriptive statistics (Observations = 18,496; Population size = 123,529,025 occupied housing units).

Variables	Population Frequency		Population Proportion	
	Count	Std. Err.	Mean	Std. Err.
***Energy assistance variables***				
Energy assistance recipient (all years)	8,670,649	305,241.90	0.0702	0.0025
Energy assistance recipient (2020)	7,015,107	273,369.80	0.0568	0.0022
Energy assistance recipient (2019)	3,698,703	211,908.90	0.0299	0.0017
Energy assistance recipient (2018)	2,860,145	191,462.60	0.0232	0.0015
Energy assistance recipient (2017)	2,268,538	171,288.70	0.0184	0.0014
Energy assistance recipient (2016)	1,988,514	152,983.50	0.0161	0.0012
Energy insecure, assistance (2020)	5,378,683	225,009.90	0.0435	0.0018
Energy insecure, no assistance (2020)	28,300,000	494,084.70	0.2290	0.0040
***Energy insecurity variables***				
Any energy insecurity	33,700,000	495,530.60	0.2725	0.0040
Broken equipment	7,269,902	276,626.00	0.0589	0.0022
Forgo necessities	24,600,000	413,548.80	0.1992	0.0033
Unhealthy temperatures	12,200,000	359,476.20	0.0987	0.0029
Medical attention required	1,422,231	93,192.99	0.0115	0.0008
Disconnection notice	12,400,000	290,964.50	0.1000	0.0024
Disconnected	2,315,706	150,672.30	0.0187	0.0012
***Respondent characteristics***				
*Income*				
Less than $20,000	18,700,000	385,608.80	0.1517	0.0031
$20,000-$39,999	24,100,000	477,498.00	0.1949	0.0039
$40,000-$59,999	19,600,000	359,684.40	0.1587	0.0029
$60,000-$99,999	27,700,000	438,701.00	0.2243	0.0036
More than $100,000	33,400,000	369,266.70	0.2704	0.0030
*Race/ethnicity*				
Black	13,000,000	288,524.30	0.1048	0.0023
Other	10,600,000	244,983.80	0.0857	0.0020
Hispanic	14,000,000	349,890.40	0.1132	0.0028
White	100,000,000	365,656.10	0.8094	0.0030
*Educational attainment*				
Less than HS diploma	5,945,443	236,789.50	0.0481	0.0019
HS diploma or GED	27,200,000	470,103.50	0.2202	0.0038
Some college or Associate's	36,900,000	512,097.00	0.2987	0.0041
Bachelor's	30,900,000	484,365.50	0.2503	0.0039
Master's, Professional, or Doctoral	22,600,000	457,238.40	0.1826	0.0037
*Household Composition*				
Number of children in household	62,200,000	1,021,709.00	0.5038	0.0083
Number of elderly in household	65,100,000	727,444.90	0.5272	0.0059
Medical device	16,900,000	353,680.90	0.1367	0.0029
Not employed	16,000,000	350,700.80	0.1298	0.0028
Retired	39,200,000	426,607.80	0.3175	0.0035
Female	67,400,000	466,744.30	0.5457	0.0038
No internet	8,804,025	232,256.80	0.0713	0.0019
***Housing characteristics***				
*Housing tenure & type*				
Renter	39,400,000	351,114.70	0.3187	0.0028
Homeowner	82,900,000	348,389.00	0.6713	0.0028
Single family home	84,500,000	23.24	0.6842	0.0000
Apartment	32,200,000	7.71	0.2605	0.0000
Mobile home	6,832,499	0.09	0.0553	0.0000
*Housing conditions*				
Poor insulation	24,900,000	401,144.80	0.2017	0.0032
Adequate insulation	98,600,000	401,145.70	0.7983	0.0032
Drafty	68,200,000	484,683.50	0.5518	0.0039
Built before 1980	63,800,000	5.42	0.5167	0.0000
Bult after 1980	59,700,000	2.29	0.4833	0.0000
Air conditioner	110,000,000	378,134.80	0.8865	0.0031
Space heater	118,000,000	255,147.90	0.9531	0.0021
Electricity use, in kwh	–	–	10565.8000	39.8228
Electricity cost	–	–	1380.0280	5.3825
***Population density***				
Urban	100,000,000	284,509.10	0.8131	0.0023
Rural	23,100,000	284,507.60	0.1869	0.0023
***Climate***				
Heating degree days	–	–	3729.1060	4.6811
Cooling degree days	–	–	1693.3770	3.6615

All variables in Table 1 are sourced from the 2020 RECS and measured in respondent *r* in state *s* [2]. Descriptions of the energy assistance and insecurity measures are provided below and more complete details regarding variable construction are available in Appendix A [80].

### 4.1 Energy assistance measures

As noted in Table 1, 7.02% of RECS respondents participated in energy bill assistance. I categorize a household as an energy assistant recipient when the respondent answered “yes” to at least one of four survey questions, including 1) “Including times when you received assistance for heating and cooling bills, has your household [ever] participated in a home energy assistance program that helps to pay energy bills or fix broken equipment?” I additionally identify households as energy assistance recipients if they respond “yes” to components of three multipart questions asking about events in the last 12 months. The three remaining questions are: 2) “When you received that [disconnection] notice, did your household apply for and receive home energy assistance to help pay your energy bill”; 3) “When that happened [cooling equipment broke], did your household apply for and receive home energy assistance to help restore your cooling?”; and 4) “When that happened [heating equipment broke], did your household apply for and receive home energy assistance to help restore your heating?” This generates a bivariate measure indicating receipt of energy assistance (“1”) or not (“0”).

Following the first energy assistance survey question noted above, respondents were asked, “In which of the following years did your household participate in a home energy assistance program? Please select all that apply.” Respondents could choose 2016, 2017, 2018, 2019, or 2020; therefore, 2016, 2017, 2018, and 2019 energy assistance recipients were coded as “1” based on which year they selected. If a respondent selected 2020 or answered “yes” to questions labeled 2, 3, or 4 above, I categorized the household as a 2020 energy assistance recipient because they explicitly ask about conditions in the past 12 months. As seen in Fig. 2, the proportion of survey respondents participating in energy assistance trends upwards.

### 4.2 Energy insecurity measures

RECS reports several energy insecurity conditions, all of which were coded as bivariate measures with “1” representing an affirmative response. First, 5.89% of respondents that responded “yes” to either of the following survey questions were coded as having broken equipment: “Your air conditioning equipment or other cooling equipment was broken, and you couldn't afford to pay for the repair or replacement” or “Your heating equipment was broken, and you couldn't afford to pay for the repair or replacement.” Second, those that responded almost every month, some months, or 1–2 months to the following three questions were, respectively, categorized as needing to forgo necessities to pay an energy bill (19.92%), keeping their home at unhealthy or unsafe temperatures (9.87%), or receiving a disconnection notice (10.00%): “In the past year, how many months did your household reduce or forego expenses for basic household necessities, such as medicine or food, in order to pay an energy bill?”; “In the past year, how many months did your household keep your home at a temperature that you felt was unsafe or unhealthy?”; or “In the past year, how many months did your household receive a disconnection notice, shut off notice, or nondelivery notice for an energy bill?”

Third, the 1.15% of households requiring medical attention due to unsafe indoor temperatures answered “yes” to either of the following questions: 1) “In the past year, did anyone in your household need medical attention because your home was too cold?” and 2) “In the past year, did anyone in your household need medical attention because your home was too hot?” Fourth, 1.87% of households reported being disconnected from their utility provider based on the following sub-questions: 1) “You couldn't pay for your electricity and it was disconnected [for heating or cooling equipment]”; 2) “You couldn't pay for your natural gas and it was disconnected [for heating equipment]”; 3) “You ran out of fuel oil, propane, wood, or pellets because you couldn't afford a delivery”; or 4) “[My power went out because I was] unable to pay electric bills.” Consequently, I constructed a measure identifying households as experiencing any type of energy insecurity in the last 12 months if at least one of the aforementioned energy insecurity measures were coded as “yes” (i.e., “1”).

Finally, I construct variables indicating if a respondent reported any measure of energy insecurity and whether they received energy assistance in 2020 or not. To do so, respondents that were coded as “1” for both experiencing any type of energy insecurity and a 2020 energy assistance recipient were identified as households that were energy insecure and received assistance. By contrast, those that were coded as “1” for experiencing any type of energy insecurity but “0” for “2020 energy assistance recipient” were categorized as households that were energy insecure but did not receive assistance.

**Fig. 3:**
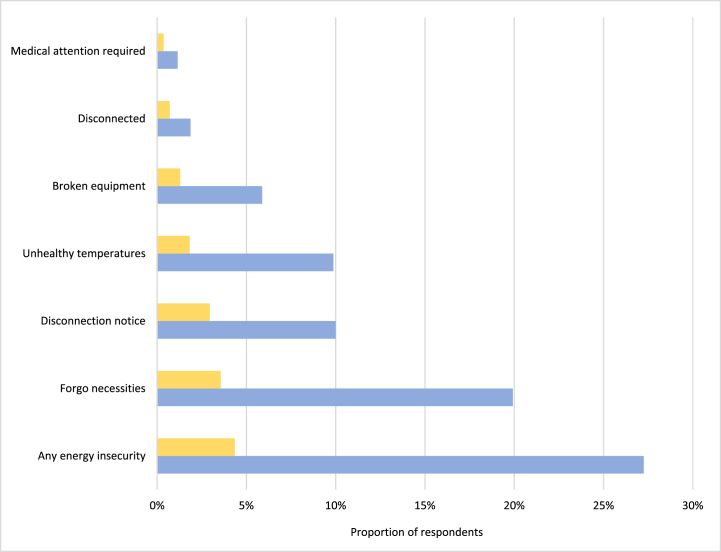
Weighted proportion of energy insecure households and energy insecure households that received assistance in 2020, according to the 2020 RECS (Observations = 18,496; Population size = 123,529,025 occupied housing units) Note: Measures are weighted proportions, calculated using 60 jackknife replications.

To learn more, I explore how often those that reported energy insecure conditions participated in energy assistance. Through Fig. 3, I compare the proportions of survey respondents that reported each type of energy insecurity (blue bar), to those that report the respective energy insecure condition and receive assistance in 2020 (yellow bar).

Fig. 3 reveals discrepancies between those that report insecurity and those that receive bill assistance in 2020. For example, 27.3% of respondents experienced at least one kind of energy insecurity; however, 4.4% of those that reported energy insecure conditions received assistance to mitigate the hardship. This suggests approximately 16% of all energy insecure households received energy bill assistance in 2020, which confirms extant literature that documents 16 to 22% of income-eligible households participate in government-sponsored energy assistance [17].

Fig. 3 disaggregates energy insecurity, illustrating a consistent pattern: more households report experiencing an energy insecure condition than those that receive assistance in 2020. Those disconnected from their utility provider – 37.9% – participated in assistance more often than those experiencing other energy insecure conditions.

### 4.3 Data limitations

The RECS has limitations to consider. First, the 2020 RECS collected data during the COVID-19 pandemic, which included an economic shock that impacted employment and purchasing power [96]; temporary financial support provided by the federal government and local entities through stimulus packages; and statewide utility disconnection moratoriums that varied by state and month [81,82]. These factors may account for the increase in participation in energy assistance between 2016 and 2020 and additionally may impact residential energy insecurity measures. Thus, the external validity of the present analysis to previous or forthcoming years is limited.

Second, I cannot account for within state variation in energy assistance participation or energy insecure conditions. Moreover, I do not know what month a household received assistance in a given year or if a household experienced energy insecure conditions outside of the study period. For this reason, I cannot disentangle within-year causality between receipt of energy assistance and energy insecure conditions. For instance, it would be beneficial to know if respondents experienced energy insecure conditions prior to the study period, allowing analyses that explored if previous energy insecure conditions predicted participation in future energy assistance. Instead, to predict receipt of assistance in 2020, I rely on energy insecure conditions as measured in 2020. This may lead to endogeneity bias and biased logistic regression estimates. I additionally cannot account for differential respondent behaviors between or within states when seasonal or weather-based electric utility protections are in place [see e.g., [[83], [84]]]. Similar limitations were noted when using the RECS in previous manuscripts [30]. For all these reasons, estimates should be interpreted as associations, rather than causal.

Third, RECS respondents are asked to recall events over the past 12 months, which introduces the threat of response and recall bias; however, there is little reason to believe systematic biases across respondents exist [59]. Lastly, the RECS does not specify the type of energy bill assistance a respondent received in its questionnaire; therefore, I do not know if a household participated in LIHEAP, utility-based programs, payment plans, or local opportunities [see Ref. [97] for an overview of energy assistance options]. Thus, concluding policy implications cannot be directed towards specific programs; instead, they will be broadly applicable to U.S. energy bill assistance programs.

### 4.4 Empirical specification

To answer the first part of research question one – identifying the correlates of energy assistance recipients – I employ weighted logistic regressions with the following model:

$$Logit\left(P\left(EA_{i}=1\right)\right)=\beta_{0}+\beta_{1}EI_{i}+B_{2}X_{i}+\gamma_{s}+\varepsilon_{i}$$

Where $P\left(EA_{i}=1\right)$ is the probability of *EA* – a binary outcome – being 1; $Logit\left(P\left(EA_{i}=1\right)\right)$ represents the natural logarithm of the odds that respondent *i* received energy assistance at least one time between 2016 and 2020*; EI* represents a vector of responses to each energy insecurity variable described above for each respondent *i*; *X* is a vector of respondent socioeconomic indicators and housing conditions (i.e., covariates), γ represents state-level fixed effects (i.e., state indicator variables leveraged from the 2020 RECS’ state-level identifiers), and ε is the error term.

Covariates are selected based on documented measures in the literature associated with energy insecure conditions. Respondent-level covariates include sex; employment status (e.g., employed, not employed, or retired); educational attainment; income in the last 12 months, collapsed into five categories: less than $20,000; $20,000-$39,999; $40,000-$59,999; $60,000-$99,999; and more than $100,000; and race and ethnicity indicators – Black, white, Hispanic, or ‘Other’ if the respondent indicated American Indian, Alaska Native, Asian, Native Hawaiian or Other Pacific Islander, or two more races [[30], [53], [85]]. I include measures for the number of children under 18 and the number of elderly (65 years or more) in a household; if the household has internet; and if anyone in the home relies on an electronic medical device [[4], [7], [86], [87]].

Housing-level covariates in the model include tenure (i.e., tenant or owner), dwelling type (i.e., single family home, apartment, or mobile home), and housing conditions – whether the home feels drafty; has poor or adequate insulation; has air conditioning; relies on a space heater; and if the home was built before or after 1980 [38,59]. I include a households' total electricity cost – in dollars – and use – in kilowatt-hours (kwh) in 2020 because energy consumption has been linked with household characteristics, including racial makeup and housing tenure [88]. Lastly, I add whether the location of the respondent's home is considered urban or rural and the number of heating and cooling degree days experienced in the last 12 months [89].

To address the second part of research question one – is receipt of energy assistance in a previous year a determinant of participation – I add previous receipt of assistance to the original empirical specification and employ logistic regression to run the following model:

(1) $$Logit\left(P\left(2020EA_{i}=1\right)\right)=\beta_{0}+\beta_{1}EA_{it}+B_{2}EI_{i}+B_{3}X_{i}+\gamma_{s}+\varepsilon_{i}\text{,}$$

Where $P\left(2020EA_{i}=1\right)$ is the probability of 2020 EA – a binary outcome – being 1; $Logit\left(P\left(2020EA_{i}=1\right)\right)$ represents the natural logarithm of the odds that respondent *I* received energy assistance in 2020*; EA* represents receipt of energy assistance for respondent *i* in year *t* for years 2016, 2017, 2018, and 2019. Although the 2020 RECS is a cross-sectional survey, I include year *t* for energy assistance measures in this and the following equation because – as noted in section 4 – one of the survey questions asks respondents to recall if they ever received energy assistance and gives them 2016, 2017, 2018, and 2019 as options. This allows me to include measures for household receipt of energy assistance over time. *EI* represents a respondent's answer to each energy insecurity condition as described above, *X* is the same vector of covariates, γ represents state-level fixed effects, and ε is the error term.

Next, to examine the second research question, I investigate how previous receipt of energy assistance impacts energy insecure conditions in 2020. To do so, I, once again, estimate logistic regressions using the following model:

(2) $$Logit\left(P\left(EI_{i}=1\right)\right)=\beta_{0}+\beta_{1}EA_{it}+B_{2}E{IR}_{i}+B_{3}X_{i}+\gamma_{s}+\varepsilon_{i}\text{,}$$

Where $P\left(EI_{i}=1\right)$ is the probability of *EI* – a series of binary outcomes – being 1; $Logit\left(P\left(EI_{i}=1\right)\right)$ represents the natural logarithm of the odds that respondent *i* responded ‘yes’ to energy insecurity measures as defined above; *EA* represents receipt of energy assistance for respondent *i* in year *t* for years 2016, 2017, 2018, and 2019; *EIR* represents a respondent's answer to the remaining energy insecurity questions not identified as the outcome measure, *X* is the same vector of covariates, γ represents state-level fixed effects, and ε is the error term.

Last, I modify the original specification to investigate the socioeconomic and housing correlates of respondents that report energy insecure conditions and receive or do not receive energy assistance in 2020. I employ the following model:

(3) $$Logit\left(P\left(E{IEA}_{i}=1\right)\right)=\beta_{0}+B_{1}X_{i}+\gamma_{s}+\varepsilon_{i}\text{,}$$

Where $P\left(E{IEA}_{i}=1\right)$ is the probability of *EIEA* – a binary outcome – being 1; $Logit\left(P\left(E{IEA}_{i}=1\right)\right)$ represents the natural logarithm of the odds that respondent *i* are categorized in the following bivariate measures: 1) respondents that report energy insecure conditions and received energy assistance and 2) respondents that report energy insecure conditions and did not receive energy assistance – for each respondent *i* in 2020*; X* is, once again, the same vector of covariates, γ represents state-level fixed effects, and ε is the error term.

## 5 Results

First, I present descriptive results and subsequently show logistic regression estimates, allowing me to examine if certain groups are associated with a differential likelihood of receiving energy assistance or experiencing energy insecure conditions, after controlling for participation in energy assistance.

### 5.1 Descriptive results: prevalence of energy assistance and insecurity

According to the 2020 weighted RECS estimates, 8,670,649 American households – 7.02% of the population – received energy bill assistance at least once between 2016 and 2020. By contrast, 27.25% of the population – 33.7 million households – reported experiencing any type of energy insecurity in 2020, revealing a gap between those that require help and those that seek out assistance to mitigate the insecurity. Therefore, to begin answering the first research question, I briefly investigate if the prevalence of energy insecure conditions and energy assistance recipients vary by characteristics in Table 2.

**Table 2:** Weighted prevalence of total U.S. households, energy insecure respondents, and energy assistance recipients in the 2020 RECS, by respondent and housing characteristics (Observations = 18,496; Population size = 123,529,025 occupied housing units).

Variables examined	Population total		Energy insecure respondent		Energy assistance recipient	
	(1) Frequency	(2) Proportion	(3) Frequency	(4) Proportion	(5) Frequency	(6) Proportion
*Respondent characteristics*						
*Income*						
Less than $20,000	18,700,000	0.1517	9,682,456	0.2876	4,286,758	0.4944
$20,000-$39,999	24,100,000	0.1949	9,565,001	0.2841	2,604,718	0.3004
$40,000-$59,999	19,600,000	0.1587	5,749,779	0.1708	920,398	0.1062
$60,000-$99,999	27,700,000	0.2243	5,596,213	0.1662	562,049	0.0648
More than $100,000	33,400,000	0.2704	3,073,229	0.0913	296,726	0.0342
*Race/ethnicity*						
Black	13,000,000	0.1048	6,744,076	0.2003	2,190,184	0.2526
Other	10,600,000	0.0857	3,693,146	0.1097	745,381	0.0860
Hispanic	14,000,000	0.1132	6,598,880	0.1960	1,383,829	0.1596
White	100,000,000	0.8094	23,200,000	0.6900	5,735,085	0.6614
*Educational attainment*						
Less than HS diploma	5,945,443	0.0481	3,053,352	0.0907	1,157,911	0.1335
HS diploma or GED	27,200,000	0.2202	9,607,598	0.2854	2,726,043	0.3144
Some college or Associat’'s	36,900,000	0.2987	12,100,000	0.3583	3,303,869	0.3810
Bachelo’'s	30,900,000	0.2503	5,950,298	0.1767	1,058,746	0.1221
Maste’'s, Professional, or Doctoral	22,600,000	0.1826	2,994,208	0.0889	424,080	0.0489
*Household Composition*						
Number of children in household	62,200,000	0.5038	24,200,000	0.7178	6,956,179	0.8023
Number of elderly in household	65,100,000	0.5272	11,700,000	0.3463	3,449,574	0.3978
Medical device	16,900,000	0.1367	5,283,386	0.1569	1,821,890	0.2101
Not employed	16,000,000	0.1298	7,743,798	0.2300	2,885,493	0.3328
Retired	39,200,000	0.3175	7,268,983	0.2159	2,456,214	0.2833
Female	67,400,000	0.5457	21,600,000	0.6421	6,239,944	0.7197
No internet	8,804,025	0.0713	2,911,373	0.0865	1,168,190	0.1347
*Housing characteristics*						
*Housing tenure & type*						
Renter	39,400,000	0.3187	16,300,000	0.4853	4,872,739	0.5620
Homeowner	82,900,000	0.6713	17,000,000	0.5042	3,676,059	0.4240
Single family home	84,500,000	0.6842	19,200,000	0.5715	4,511,316	0.5203
Apartment	32,200,000	0.2605	11,200,000	0.3332	3,186,093	0.3675
Mobile home	6,832,499	0.0553	3,206,498	0.0952	973,240	0.1122
*Housing conditions*						
Poor insulation	24,900,000	0.2017	11,700,000	0.3473	3,500,791	0.4038
Adequate insulation	98,600,000	0.7983	22,000,000	0.6527	5,169,858	0.5962
Drafty	68,200,000	0.5518	24,200,000	0.7191	6,748,760	0.7783
Built before 1980	63,800,000	0.5167	19,100,000	0.5682	5,469,109	0.6308
Bult after 1980	59,700,000	0.4833	14,500,000	0.4318	3,201,540	0.3692
Air conditioner	110,000,000	0.8865	28,600,000	0.8480	7,247,370	0.8359
Space heater	118,000,000	0.9531	31,500,000	0.9344	8,272,701	0.9541
Electricity use, in kwh	–	10,565.80	–	10,530.92	–	9846.29
Electricity cost	–	1380.03	–	1381.81	–	1285.76
*Population density*						
Urban	100,000,000	0.8131	28,100,000	0.8353	7,434,056	0.8574
Rural	23,100,000	0.1869	5,543,547	0.1647	1,236,593	0.1426

Notes: 1) Columns 1, 3, and 5 contain weighted frequency values; 2) Columns 2, 4, and 6 contain weighted proportions; and 3) measures in all columns were calculated using 60 jackknife replications.

Table 2 shows the total weighted frequency and proportion of U.S. households across respondent and housing characteristics (columns 1 and 2), energy insecure households (columns 3 and 4), and energy assistance recipients (columns 5 and 6). As expected, Table 2 illustrates that several groups experience energy insecure conditions and receive energy assistance at higher rates than others. For example, Black and Hispanic respondents are, respectively, 10.5 and 11.3% of the total survey population, but approximately 20% of both groups experience energy insecurity and 25.3 and 16% of Black and Hispanic respondents, respectively, receive energy assistance. I also find those with less than $60,000 annual incomes are 50.5% of the population, but 74.25 and 90.1% experience energy insecurity and receive assistance, respectively. Additionally, 71.8% of children experience energy insecurity in their homes and 80.2% receive energy assistance, but they are only 50.4% of the survey population. Lastly, respectively, 31.9, 20.2, and 55.2% of the survey population rent their homes, live in homes with poor insulation, and have drafty indoor conditions; however, 48.5, 34.7, and 71.9% of these households report energy insecurity and 56.2, 40.4, and 77.8% received energy assistance.

Table 2 reveals additional disparities for respondents that require medical devices, are not employed, and live in homes built before 1980. Lastly, I find that the mean amount of electricity used by those participating in energy assistance is 719.51 kwh less than the full population, suggesting that those that require financial assistance to pay an energy bill may be simultaneously limiting their household energy consumption.

### 5.2 Regression results: correlates of energy assistance recipients

To answer the first research question, I present Table 3, which identifies the correlates of energy assistance recipients. Column 1 addresses the first part of research question one, revealing the indicators associated with respondents that received energy assistance at least once between 2016 and 2020. Column 2 addresses the second part of research question one, examining indicators – including previous receipt of assistance – associated with receipt of energy assistance in 2020.

**Table 3:** Correlates of energy assistance recipients.

VARIABLES	(1)	(2)
	Energy assistance recipient	2020 energy assistance recipient
*Energy assistance variables*		
2019 energy assistance recipient		54.46**
		(16.103)
2018 energy assistance recipient		3.14**
		(1.083)
2017 energy assistance recipient		1.46
		(0.685)
2016 energy assistance recipient		2.92*
		(1.069)
*Energy insecurity variables*		
Broken heating or AC	1.57**	1.98**
	(0.177)	(0.314)
Forgo necessities	1.78**	1.64*
	(0.172)	(0.266)
Unhealthy temperature	1.12	1.35+
	(0.119)	(0.207)
Medical attention required	1.29	1.17
	(0.322)	(0.356)
Disconnection notice	4.19**	10.23**
	(0.505)	(1.669)
Disconnected	1.47+	1.87*
	(0.286)	(0.373)
*Respondent characteristics*		
Less than $20,000	13.71**	8.16**
	(3.335)	(3.039)
$20,000-$39,999	6.93**	4.57**
	(1.578)	(1.626)
$40,000-$59,999	3.21**	2.69*
	(0.723)	(0.949)
$60,000-$99,999	1.73*	1.51
	(0.368)	(0.520)
Black	1.78**	1.47+
	(0.233)	(0.280)
Other race	0.92	1.03
	(0.157)	(0.243)
Hispanic	0.87	0.84
	(0.102)	(0.147)
Less than HS	1.47*	1.09
	(0.231)	(0.307)
HS diploma/GED	1.14	0.96
	(0.139)	(0.175)
Some college/Associate's	1.46**	1.19
	(0.165)	(0.190)
Children under 18	1.14**	1.11+
	(0.043)	(0.057)
Elderly	0.93	0.86
	(0.066)	(0.096)
Medical device	1.54**	1.33
	(0.194)	(0.230)
Not employed	1.44**	1.83**
	(0.155)	(0.290)
Retired	1.24	1.53*
	(0.170)	(0.286)
Female	1.45**	1.31+
	(0.122)	(0.178)
No internet	1.39*	1.42
	(0.188)	(0.312)
*Housing characteristics*		
Renters	1.18	1.31
	(0.150)	(0.232)
Apartment	0.90	0.88
	(0.115)	(0.176)
Mobile home	1.13	1.21
	(0.184)	(0.259)
Poor or no insulation	1.12	0.97
	(0.109)	(0.131)
Drafty	1.40**	1.06
	(0.115)	(0.150)
Built before 1980	1.12	1.02
	(0.130)	(0.174)
Air conditioning	1.06	0.70+
	(0.122)	(0.120)
Space heater	1.80+	2.43+
	(0.496)	(1.019)
Electricity use, in kwh	1.00**	1.00*
	(0.000)	(0.000)
Electricity cost	1.00**	1.00
	(0.000)	(0.000)
*Population density*		
Urban	1.30+	1.70*
	(0.170)	(0.342)
*Climate*		
Heating degree days	1.00*	1.00
	(0.000)	(0.000)
Cooling degree days	1.00+	1.00
	(0.000)	(0.000)
Constant	0.00**	0.00**
	(0.000)	(0.000)
State FE	Yes	Yes
Observations	18,496	18,496

Notes: 1) Columns contain weighted logistic regression estimates (i.e., odds ratios), with 60 jackknife replicate standard errors in parentheses; 2) Levels of significance: **p < 0.01, *p < 0.05, + p < 0.1; and 3) Omitted categories include: respondents with $100,000 or more income; white respondents; respondents with a Bachelor's, Master's, Professional, or Doctoral degree; homeowners; single family homes – detached or attached; adequate insulation; built after 1980; and rural.

Supporting expectations for both parts of research question one, Table 3 illustrates several indicators predict receipt of energy assistance. Column 1 reveals many energy insecure measures – those who reported broken heating or cooling equipment, need to forgo necessities to pay an energy bill, received a disconnection notice, and were disconnected from their utility provider – have a higher odds than those that did not experience these conditions to receive assistance any time between 2016 and 2020. Column 2 shows these measures and households that experience unhealthy indoor temperatures are associated with receipt of assistance in 2020, when controlling for previous receipt of assistance. Among the insecurity measures, those that received a disconnection notice in 2020 had the highest odds of ever receiving assistance and receiving it in 2020.

Column 2 further supports expectations, reporting respondents that received energy assistance in 2019, 2018, and 2016 have, respectively, a 54.46, 3.14, and 2.92 higher odds of receiving assistance in 2020 than those that did not receive assistance in those years. The relatively large odds ratios – especially the estimated coefficient for a 2019 energy assistant recipient – indicate previous receipt of assistance is likely a substantively important determinant of participation in energy assistance.

I find several populations have higher odds of receiving assistance in both models, indicating these characteristics remain statistically significant even when controlling for previous receipt of assistance. Respondents with incomes less than $60,000, as compared to those with incomes over $100,000; Black respondents; households with more children under 18; respondents that are not employed, female, use a space heater; those that use more electricity (in kwh); and live in urban areas are statistically associated with receipt of assistance in both models.

Moreover, when I control for previous receipt of assistance, several respondent and housing characteristics lose significance (i.e., indicators are significant in column 1 but not in column 2). Households without internet; with incomes between $60,000-$99,999; those that rely on an electronic medical device; all education indicators; those that live in drafty conditions and have higher electricity costs; and living in areas with more heating degree days are more likely to receive assistance at least one time between 2016 and 2020; however, these groups are no more or less likely to receive assistance specifically in 2020 after controlling for previous receipt of assistance. Lastly, retired respondents are only statistically associated with receipt of assistance in 2020 (column 2).

The lack of a statistical association between receipt of assistance and Hispanic respondents, renters, and those with poor insulation are contrary to expectations and noteworthy null findings, suggesting that these groups are not more or less likely to participate in formal assistance despite being previously documented as energy insecure.

### 5.3 Regression results: correlates of energy insecure respondents

**Fig. 4:**
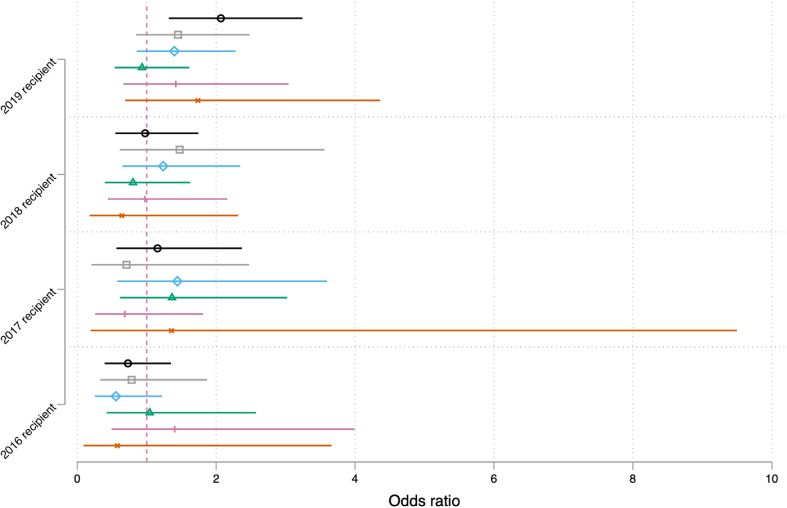
Energy assistance predictors of energy insecurity measures Notes: 1) Values are weighted logistic regression estimates (i.e., odds ratios) for previous energy assistance recipients with 95 % CIs, estimated from models that include all covariates, state FEs, and 60 jackknife replicate standard errors; 2) CIs are wider for disconnection because they are rarer events.

Next, I answer the second research question by exploring if receipt of energy bill assistance in previous years – 2019, 2018, 2017, 2016 – is associated with a respondent's odds of experiencing energy insecurity in 2020. Fig. 4 displays the results of logistic regression models predicting if previous receipt of assistance is associated with any household energy insecurity (black circle), broken equipment (grey square), the need to forgo necessities (blue diamond), keeping unhealthy indoor temperatures (green triangle), receiving a disconnection notice (pink line), and being disconnected (orange cross).^2^

Fig. 4 reports that previous receipt of energy assistance does not reduce the odds of a respondent experiencing energy insecurity. Instead, receipt of assistance in 2018, 2017, and 2016 is not statistically associated with any energy insecurity measure reported in 2020. Yet, receipt of assistance in 2019 is associated with an increased odds of a respondent reporting any energy insecurity measure in 2020. This outcome is expected and corroborates Memmott et al. (2021)'s [7] finding that receipt of any form of government assistance is associated with a higher risk of experiencing energy insecurity.^3^

The remaining respondent and housing characteristic regression estimates are available in Table 4, revealing race, educational attainment, having children in the home, housing tenure and conditions, and reliance on an electronic medical device are commonly associated with energy insecure conditions, even when controlling for household income and prior participation in energy assistance. Specifically, estimates in Table 4 column 1 reveal Black and Hispanic respondents, those with less than a college education, households with children and elderly members, that rely on an electronic medical device, are female, tenants, live in mobile homes, have deficient housing conditions, live in a house built before 1980, and have higher electricity costs are more likely to experience any type of energy insecurity in 2020, after controlling for income and previous energy assistance participation (i.e., receipt of energy assistance in 2019, 2018, 2017, and/or 2016).

**Table 4:** Remaining correlates of energy insecurity measures, controlling for prior participation in energy assistance.

VARIABLES	(1)	(2)	(3)	(4)	(5)	(6)
	Any energy insecurity	Broken equipment	Forgo necessities	Unhealthy temperatures	Disconnection notice	Disconnected
*Energy insecurity variables*						
Broken heating or AC			2.83**	2.76**	1.69**	1.63+
			(0.314)	(0.299)	(0.206)	(0.349)
Forgo necessities		2.94**		5.91**	6.04**	3.83**
		(0.314)		(0.564)	(0.644)	(0.953)
Unhealthy temperature		2.75**	5.64**		1.65**	2.10**
		(0.298)	(0.516)		(0.183)	(0.383)
Medical attention required		3.50**	1.28	4.60**	1.46	2.23*
		(0.857)	(0.418)	(1.192)	(0.367)	(0.645)
Disconnection notice		1.74**	5.71**	1.57**		8.30**
		(0.212)	(0.595)	(0.167)		(1.650)
Disconnected		1.49	2.69**	1.91**	8.19**	
		(0.321)	(0.634)	(0.366)	(1.671)	
*Respondent characteristics*						
Less than $20,000	8.07**	2.53**	7.31**	2.55**	1.95**	2.44+
	(0.889)	(0.525)	(1.062)	(0.395)	(0.374)	(1.094)
$20,000-$39,999	6.27**	2.88**	6.46**	2.03**	1.85**	1.81
	(0.546)	(0.549)	(0.737)	(0.263)	(0.301)	(0.694)
$40,000-$59,999	4.17**	2.73**	4.41**	1.41*	2.20**	1.28
	(0.365)	(0.529)	(0.517)	(0.192)	(0.348)	(0.502)
$60,000-$99,999	2.54**	1.75*	2.78**	1.14	1.80**	1.00
	(0.185)	(0.312)	(0.388)	(0.141)	(0.283)	(0.386)
Black	2.48**	1.42*	1.67**	1.00	2.31**	1.38
	(0.183)	(0.188)	(0.140)	(0.122)	(0.208)	(0.251)
Other race	1.76**	1.31	1.53**	1.19	0.97	1.15
	(0.144)	(0.221)	(0.172)	(0.171)	(0.134)	(0.321)
Hispanic	1.94**	1.02	1.71**	1.22	1.22	1.00
	(0.137)	(0.147)	(0.169)	(0.151)	(0.156)	(0.254)
Less than HS	1.61**	0.78	1.78**	0.74+	1.37+	2.26*
	(0.171)	(0.151)	(0.258)	(0.112)	(0.230)	(0.550)
HS diploma/GED	1.32**	0.87	1.64**	0.70*	1.47**	1.62+
	(0.090)	(0.111)	(0.138)	(0.079)	(0.133)	(0.322)
Some college/Associate's	1.47**	1.05	1.71**	0.77*	1.44**	1.57+
	(0.101)	(0.127)	(0.119)	(0.071)	(0.153)	(0.324)
Children under 18	1.17**	1.14*	1.13**	0.89*	1.15**	1.11
	(0.034)	(0.048)	(0.036)	(0.039)	(0.046)	(0.082)
Elderly	0.76**	0.88	0.77**	1.04	0.80*	0.86
	(0.032)	(0.069)	(0.046)	(0.077)	(0.062)	(0.150)
Medical device	1.38**	1.26+	1.15	1.01	1.48**	0.82
	(0.086)	(0.136)	(0.091)	(0.104)	(0.144)	(0.216)
Not employed	1.00	0.82	0.94	0.93	1.19	1.05
	(0.072)	(0.094)	(0.082)	(0.103)	(0.120)	(0.192)
Retired	0.61**	0.63**	0.67**	0.92	0.67**	0.62
	(0.048)	(0.082)	(0.055)	(0.114)	(0.079)	(0.174)
Female	1.18**	1.04	1.11	0.82*	1.45**	0.70+
	(0.052)	(0.100)	(0.075)	(0.055)	(0.131)	(0.110)
No internet	0.84	1.16	1.07	0.89	0.65+	1.16
	(0.090)	(0.206)	(0.125)	(0.116)	(0.134)	(0.368)
*Housing characteristics*						
Renters	1.48**	0.55**	1.24*	1.01	1.97**	0.91
	(0.099)	(0.053)	(0.102)	(0.095)	(0.184)	(0.171)
Apartment	0.79*	0.73+	1.07	1.02	0.73*	0.73
	(0.070)	(0.109)	(0.102)	(0.098)	(0.082)	(0.184)
Mobile home	1.48**	1.55*	1.19	0.95	1.61**	0.87
	(0.130)	(0.242)	(0.129)	(0.146)	(0.179)	(0.252)
Poor or no insulation	1.70**	1.51**	1.08	1.85**	1.06	1.12
	(0.097)	(0.122)	(0.082)	(0.172)	(0.091)	(0.206)
Drafty	1.67**	1.38*	1.39**	1.49**	1.40**	1.23
	(0.075)	(0.158)	(0.094)	(0.121)	(0.112)	(0.232)
Built before 1980	1.14*	1.13	1.10	0.97	1.23*	0.96
	(0.052)	(0.094)	(0.061)	(0.068)	(0.105)	(0.170)
Air conditioning	0.83*	1.08	1.01	0.75*	1.04	0.93
	(0.067)	(0.153)	(0.106)	(0.082)	(0.139)	(0.207)
Space heater	0.86	0.72	0.99	0.78	1.06	0.71
	(0.108)	(0.169)	(0.160)	(0.136)	(0.225)	(0.229)
Electricity use, in kwh	1.00	1.00	1.00	1.00*	1.00	1.00+
	(0.000)	(0.000)	(0.000)	(0.000)	(0.000)	(0.000)
Electricity cost	1.00**	1.00	1.00+	1.00	1.00+	1.00*
	(0.000)	(0.000)	(0.000)	(0.000)	(0.000)	(0.000)
*Population density*						
Urban	1.07	1.20	1.03	0.94	1.26+	0.52**
	(0.068)	(0.143)	(0.073)	(0.093)	(0.148)	(0.091)
*Climate*						
Heating degree days	1.00	1.00	1.00	1.00	1.00	1.00
	(0.000)	(0.000)	(0.000)	(0.000)	(0.000)	(0.000)
Cooling degree days	1.00	1.00	1.00	1.00	1.00	1.00
	(0.000)	(0.000)	(0.000)	(0.000)	(0.000)	(0.000)
Constant	0.03**	0.01**	0.01**	0.05**	0.00**	0.00**
	(0.011)	(0.005)	(0.005)	(0.022)	(0.001)	(0.003)
Energy assistance variables	Yes	Yes	Yes	Yes	Yes	Yes
State FE	Yes	Yes	Yes	Yes	Yes	Yes
Observations	18,496	18,496	18,496	18,496	18,496	18,496

Notes: 1) Columns contain weighted logistic regression estimates (i.e., odds ratios), with 60 jackknife replicate standard errors in parentheses; 2) Levels of significance: **p < 0.01, *p < 0.05, + p < 0.1; and 4) Omitted categories include: respondents with $100,000 or more income; white respondents; respondents with a Bachelor's, Master's, Professional, or Doctoral degree; homeowners; single family homes – detached or attached; adequate insulation; built after 1980; and rural.

Table 4 additionally shows the predictors of explicit measures of energy insecurity in columns 2 through 6. First, column 2 illustrates Black respondents, those that use an electronic medical device, have children in the home, live in mobile homes, and with deficient housing conditions are more likely to have broken equipment. Second, column 3 indicates Black and Hispanic respondents, those that identify as a race other than Black, Hispanic, or white, those with less than a college education, households with children, drafty housing conditions, and tenants are more likely to forgo necessities, such as food and medicine, to pay an energy bill. Third, as seen in column 4, higher electricity use is correlated with homes keeping their indoor temperatures at uncomfortable or unsafe levels due to energy cost concerns, as do homes with poor or no insulation and drafty conditions. Notably, homes with more children under 18 are less likely to keep their homes at unhealthy or unsafe temperatures indicating that caregivers may prioritize indoor thermal comfort to keep children safe.

Fourth, column 5 reveals several groups have a higher odds of receiving a disconnection notice from their utility provider. They include Black and female respondents, tenants, those with less than a college education, those with more children, and those relying on an electronic medical device. Households that receive disconnection notices are also more likely to live in drafty conditions, mobile homes, homes built before 1980, have higher electricity costs, and in urban areas. Lastly, column 6 indicates those with less than a college degree as well as those with higher electricity use and costs had a higher odds of being disconnected from their utility providers, even when controlling for income and previous participation in energy assistance. Markedly, female respondents and those living in urban areas are less likely to be disconnected from the grid.

The final set of results investigates the correlates of those that reported being energy insecure and received assistance as well as those that reported being energy insecure but did not receive assistance in 2020. Table 5 displays the estimates from logistic regression models.

**Table 5:** Correlates of energy insecure respondents that received and did not receive energy assistance in 2020.

VARIABLES	(1)	(2)
	Insecure, received assistance	Insecure, no assistance
*Respondent characteristics*		
Less than $20,000	35.92**	4.51**
	(12.305)	(0.480)
$20,000-$39,999	16.27**	4.83**
	(5.596)	(0.383)
$40,000-$59,999	9.45**	3.54**
	(3.112)	(0.287)
$60,000-$99,999	3.14*	2.42**
	(1.098)	(0.161)
Black	2.33**	1.91**
	(0.313)	(0.139)
Other race	1.12	1.75**
	(0.185)	(0.133)
Hispanic	1.02	1.98**
	(0.150)	(0.141)
Less than HS	1.61*	1.44*
	(0.299)	(0.162)
HS diploma/GED	1.18	1.32**
	(0.191)	(0.090)
Some college/Associate's	1.52*	1.40**
	(0.221)	(0.095)
Children under 18	1.25**	1.07*
	(0.047)	(0.032)
Elderly	0.77*	0.79**
	(0.082)	(0.034)
Medical device	1.79**	1.17*
	(0.290)	(0.080)
Not employed	1.46*	0.85+
	(0.177)	(0.064)
Retired	0.88	0.62**
	(0.140)	(0.049)
Female	1.51*	1.09+
	(0.186)	(0.048)
No internet	1.18	0.84
	(0.222)	(0.092)
*Housing characteristics*		
Renters	1.32	1.29**
	(0.214)	(0.084)
Apartment	0.78	0.90
	(0.117)	(0.074)
Mobile home	1.56*	1.30*
	(0.256)	(0.112)
Poor or no insulation	1.44**	1.51**
	(0.152)	(0.086)
Drafty	1.97**	1.53**
	(0.250)	(0.068)
Built before 1980	1.21	1.11+
	(0.155)	(0.055)
Air conditioning	0.88	0.87
	(0.121)	(0.070)
Space heater	1.42	0.79
	(0.501)	(0.109)
Electricity use, in kwh	1.00*	1.00
	(0.000)	(0.000)
Electricity cost	1.00	1.00**
	(0.000)	(0.000)
*Population density*		
Urban	1.41+	0.99
	(0.224)	(0.066)
*Climate*		
Heating degree days	1.00	1.00
	(0.000)	(0.000)
Cooling degree days	1.00+	1.00
	(0.000)	(0.000)
Constant	0.00**	0.08**
	(0.000)	(0.025)
State FE	Yes	Yes
Observations	18,496	18,496

Notes: 1) Cells contain weighted logistic regression estimates (i.e., odds ratios), with 60 jackknife replicate standard errors in parentheses; 2) Levels of significance: **p < 0.01, *p < 0.05, + p < 0.1; 3) Omitted categories include: respondents with $100,000 or more income; white respondents; respondents with a Bachelor's, Master's, Professional, or Doctoral degree; homeowners; single family homes – detached or attached; adequate insulation; built after 1980; and rural; and 4) Models do not include energy assistance or insecurity measures as covariates because these variables are embedded in outcome measures.

Table 5 reveals – once again – several respondent and housing characteristics are associated with being insecure and participating in assistance and being insecure but not participating in assistance. These include incomes below $100,000, educational attainment, Black and female respondents, households with more children, those that rely on an electronic medical device, as well as those that live in mobile homes and report deficient housing conditions. However, column 2 shows that other indicators – Hispanic, those that identify as a race other than Black or white, have a HS diploma or GED, as well as those with higher electricity costs, that rent their homes, and live in homes built before 1980 – have a higher odds of being insecure but not participating in assistance.

## 6 Conclusion and policy implications

Prominent global institutions agree adequate residential energy consumption is essential for individuals to participate in the modern economy [90] and crucial to eradicate poverty, combat climate change, and advance opportunities in health, education, and gender equity [91]. Despite its importance, scant scholarship focuses on assistance programs aiming to help U.S. individuals and families retain access to power, meet non-discretionary energy consumption demands, and pay energy bills [see e.g., [[69], [77]]]. For this reason, I rely on the EIA's 2020 RECS to investigate household characteristics associated with energy bill assistance participation in the U.S. and consider if receipt of financial assistance affects residential energy insecure conditions.

Findings of the present analysis contribute to the growing scholarly literature examining the coping mechanisms American households engage in (i.e., behavioral energy insecurity) to minimize economic and physical energy insecurity [see e.g., [[4], [5]]]. Descriptive results confirm participation in energy bill assistance is low relative to the proportion of households experiencing energy insecure conditions. While 33.7 million households endured at least one energy insecure condition in 2020, only 7 million received energy bill assistance. These estimates reinforce domestic energy assistance programs are under-resourced [6], leaving millions of American households unable to access one of the safest strategies available [92].

Empirical estimates – all of which include state-level fixed effects – indicate some populations are more likely to receive assistance than others. In answering the first research question (i.e., identifying determinants of energy assistance recipients), analysis of the 2020 RECS finds lower-income, Black individuals, those with more children under 18, elderly individuals, and those living in urban communities had higher odds of participating in energy bill assistance in 2020. However, my analysis also reveals groups documented as energy insecure in previous literature [see e.g., [7]] – Hispanic respondents, those with less than a college education, those that rely on electronic medical devices, renters, and those living with physical energy insecurity (i.e., poor or no insulation, drafty conditions, homes built before 1980) – did not have a higher likelihood of participating in assistance in 2020.

The analysis also reveals two indicators have outsized logistic regression estimates: previous participation in energy assistance and receipt of a disconnection notice from a utility provider. Associations of the two measures indicate they might have a relatively large, substantial impact on the likelihood of participating in energy bill assistance. First, the results suggest individuals who received energy assistance in 2019 were more likely to receive assistance in 2020. This finding suggests the demand for energy bill assistance remains consistent year-to-year, and those that navigated the application process in the previous year are more likely to successfully engage in assistance again. Second, if a respondent received a disconnection notice from its utility provider, they had 10 times higher odds of participating in assistance. In addition to highlighting a respondent's urgent need for financial help, the estimate reveals a potentially crucial pathway – formal communication from a utility provider – utilities and energy assistance administrators should consider when connecting energy insecure households with assistance options.

Next, to answer the second research question, investigation of the 2020 RECS find previous participation in energy assistance – in 2019, 2018, 2017, and/or 2016 – does not reduce the likelihood that a household experiences energy insecurity in 2020. In fact, receipt of assistance in 2019 is associated with an increased likelihood a household experiences any form of energy insecurity in 2020. As noted, the circular relationship of energy insecure conditions and participation in energy bill assistance as well as data limitations (e.g., lack of data on energy insecure conditions outside the 2020 RECS study period) prevent assessment of causality. Despite the limitation, this finding indicates the current financial assistance options are inadequate to help households address energy insecure conditions.

I additionally find, even after controlling for receipt of assistance and income, several household characteristics remain statistically associated with energy insecure conditions. Notably, empirical models affirm race, educational attainment, those with children in the home, tenants, living with deficient housing conditions, and those reliant on an electronic medical device continue to experience energy insecure conditions at higher rates [see e.g. [[30], [59]]]. Moreover, even after controlling for prior participation in assistance, respondents with less than a college degree retained a statistically higher odds of being disconnected. Taken in totality, these associations suggest vulnerable populations are not receiving adequate financial support from energy assistance programs to meet their energy consumption needs.

Lastly, I identify households more likely to be associated with energy insecurity yet not participate in assistance. Results indicate Hispanic respondents, tenants, and individuals residing in older homes are at a higher risk of experiencing energy insecurity, yet they do not receive assistance to alleviate the hardship. This provides an opportunity for public administrators to enhance outreach initiatives targeting these groups, as they are particularly vulnerable due to their limited engagement with energy assistance programs [see e.g., [93]].

The findings of the present analysis have three policy implications. The first implication is informed by extant literature and supported by the findings in this article, whereas the second and third implications are primarily generated from the results of this analysis. First, like other scholars, I recommend decisionmakers officially recognize American households consistently face economic and physical energy insecure conditions [see e.g., [30]]. Recognizing energy insecurity as a distinct, prevalent, and persistent material hardship – as elaborated by Bednar and Reames (2020) [33] – will allow federal, state, and local policymakers to resource and design energy bill assistance programs that will more adequately match the scope and scale of the problem that impacts over a quarter of the U.S. population. For example, in 2020, 27.8 million U.S. households were income-eligible for federal energy assistance, but only 5.6 million participated in LIHEAP [72]. Additionally, unlike SNAP, which provides monthly benefits to qualified individuals, LIHEAP and other energy assistance programs are offered annually. However, since energy expenses, like food, are incurred monthly, it is crucial for financial assistance to align with the recurring needs of households. Therefore, energy assistance should be provided in a manner that reflects the ongoing monthly requirement to consume energy and pay energy bills.

Second, through my analysis of the 2020 RECS, I identify pathways decisionmakers should consider when trying to connect energy insecure households with low-risk, publicly funded assistance opportunities. The estimate revealing a significant and substantive association between receipt of a disconnection notice and participation in energy assistance suggests formal communication from utility providers may be both prudent and effectual in connecting households in need with financial assistance options. Therefore, rather than waiting until a household is in dire need, utility providers could send formal communication to households immediately after a household's first partial or missed payment.

The data, however, masks the mechanism that allows disconnection notices to connect customers with assistance options. For example, it is unclear if it is a household's urgent need for financial relief, the disconnection notice itself, or if utilities provide instructions and contact information for available energy assistance opportunities alongside disconnection notices. Future research should aim to identify the specific mechanism by investigating utility-level practices and state-level disconnection policies. Almost all 50 states have a state disconnection policy that prevents disconnections during certain times of the year (e.g., hot summer and cold winter months) [see e.g., [83]]. These disconnection policies vary across states; therefore, scholars should inquire if states require utilities to send information about energy assistance opportunities alongside disconnection notices. Scholars could leverage the variation across states and utilities to discern if it is, in fact, the disconnection notice itself or any additional material (e.g., a flyer with local energy assistance phone number and website) that connects energy insecure households with energy assistance programs.

Third, another pathway connecting households to energy bill assistance identified in this analysis is previous receipt of assistance. This finding indicates energy assistance administrators should prioritize expanding the number of eligible households enrolled in programs by resourcing outreach efforts and targeting energy insecure households that have never participated in assistance. Administrators should direct resources towards those with less than a college degree given its vulnerability to being disconnected as well as Hispanic populations, tenants, and communities that live in older homes because these populations had a higher odds of being insecure yet not participating in assistance. Besides expansion of funding for assistance programs, this may the most effective method to increase program participation. Results suggest ensuring households previously unaware or unable to access energy assistance programs can successfully navigate the process substantially increases likelihood of future participation. For this reason, administrators should prioritize generating awareness about available programs, providing clear instruction on how to apply, and offering guidance through the application process to new applicants.

The economic and physical energy insecurity facing millions of U.S. households forcefully reminds us that the current publicly funded assistance options are inadequate to help those that cannot meet their non-discretionary energy consumption needs [94]. As the effects of climate change become ubiquitous, we expect residential financial needs to grow [95]. Therefore, decisionmakers should not only expand resources expended for these programs, energy assistance administrators and utility providers must be mindful of the pathways that connect energy insecure populations with energy assistance opportunities. Future scholarship should continue to identify and evaluate these pathways to generate a comprehensive local energy assistance framework, identifying both barriers as well as opportunities that connect energy insecure households with energy assistance opportunities;, provide important insights that will allow practitioners to attract new applicants; and expand participation in a cost-effective manner.

## Data availability

Datasets related to this article can be found at https://www.eia.gov/consumption/residential/data/2020/index.php?view=microdata, hosted at the U.S. Energy Information Administration (EIA) [2].

## Funding sources

This research did not receive any specific grant from funding agencies in the public, commercial, or not-for-profit sectors.

## Additional information

No additional information is available for this paper.

## CRediT authorship contribution statement

**Michelle Graff:** Writing – review & editing, Writing – original draft, Visualization, Validation, Supervision, Software, Resources, Project administration, Methodology, Investigation, Funding acquisition, Formal analysis, Data curation, Conceptualization.

## Declaration of competing interest

The authors declare that they have no known competing financial interests or personal relationships that could have appeared to influence the work reported in this paper.

## Figures and Tables

**Table A1:** Description and detail of variables

Variables	Measures
***Energy assistance variables***	
Energy assistance recipient	Constructed variable measured by response to survey questions: 1) “Including times when you received assistance for heating and cooling bills, has your household participated in a home energy assistance program that helps to pay energy bills or fix broken equipment?”; 2) When you received that [disconnection] notice, did your household apply for and receive home energy assistance to help pay your energy bill; 3) When that happened, did your household apply for and receive home energy assistance to help restore your cooling?; and 4) When that happened, did your household apply for and receive home energy assistance to help restore your heating?” Respondents that reported “yes” to questions 1–4, variable coded as 1.
2020 energy assistance recipient	Constructed variable measured by response to survey questions: 1) “Including times when you received assistance for heating and cooling bills, did your household participate in a home energy assistance program that helps to pay energy bills or fix broken equipment in 2020?”; 2) When you received that [disconnection] notice, did your household apply for and receive home energy assistance to help pay your energy bill; 3) When that happened, did your household apply for and receive home energy assistance to help restore your cooling?; and 4) When that happened, did your household apply for and receive home energy assistance to help restore your heating?” Respondents that reported “yes” to questions 1–4, variable coded as 1.
Energy assistance recipient – 2019 Energy assistance recipient – 2018 Energy assistance recipient – 2017 Energy assistance recipient – 2016	Measured by response to follow up first survey question described in ‘Energy assistance recipient’: “In which of the following years did your household participate in a home energy assistance program? Please select all that apply.” Respondents that reported “yes” in year *t*, variable was coded as 1.
Additive energy assistance	Constructed variable that added respondents' responses to whether they received energy assistance in 2020, 2019, 2018, 2017, and 2016. Variable ranges from 0 to 5.
Energy insecure, assistance – 2020	Constructed variable. Respondents that coded “1” to ‘Any energy insecurity’ and “1” to “2020 energy assistance recipient” was coded as 1.
Energy insecure, no assistance – 2020	Constructed variable. Respondents that coded “1” to ‘Any energy insecurity’ and “0” to “2020 energy assistance recipient” was coded as 1.
***Energy insecurity variables***	
Any energy insecurity	Constructed variable. Respondents that reported “yes” to any energy insecurity question, variable was coded as 1.
Broken equipment	Constructed variable measured by response to survey questions: 1) “Your air conditioning equipment or other cooling equipment was broken, and you couldn't afford to pay for the repair or replacement” and 2) “Your heating equipment was broken, and you couldn't afford to pay for the repair or replacement.” Respondents that reported “yes” to either or both questions, variable was coded as 1.
Forgo necessities	Measured by response to survey question: “In the past year, how many months did your household reduce or forego expenses for basic household necessities, such as medicine or food, in order to pay an energy bill?” Respondents that reported “almost every month,” “some months,” or “1–2 months,” variable was coded as 1.
Unhealthy temperatures	Measured by response to survey question: “In the past year, how many months did your household keep your home at a temperature that you felt was unsafe or unhealthy?” Respondents that reported “almost every month,” “some months,” or “1–2 months,” variable was coded as 1.
Medical attention required	Constructed variable measured by response to survey questions: 1) “In the past year, did anyone in your household need medical attention because your home was too cold?” and 2) “In the past year, did anyone in your household need medical attention because your home was too hot?” Respondents that reported “yes” to either or both questions, variable was coded as 1.
Disconnection notice	Measured by response to survey question: “In the past year, how many months did your household receive a disconnection notice, shut off notice, or nondelivery notice for an energy bill?” Respondents that reported “almost every month,” “some months,” or “1–2 months,” variable was coded as 1.
Disconnected	Constructed variable measured by response to survey questions: 1) “You couldn't pay for your electricity and it was disconnected [for heating or cooling equipment]”; 2) “You couldn't pay for your natural gas and it was disconnected [for heating equipment]”; 3) “You ran out of fuel oil, propane, wood, or pellets because you couldn't afford a delivery”; and 4) “[My power went out because] unable to pay electric bills.” Respondents that reported “yes” to any of these questions, variable was coded as 1.
***Respondent characteristics***	
*Income*	Measured by response to: “Including all income sources, what category best describes the total combined income of all household members for the past year, before taxes and deductions?”
Less than $20,000	Coded as 1 (“Less than $20,000”) and 0 (“not”).
$20,000-$39,999	Coded as 1 (“$20,000-$39,999”) and 0 (“not”).
$40,000-$59,999	Coded as 1 (“$40,000-$59,999”) and 0 (“not”).
$60,000-$99,999	Coded as 1 (“$60,000-$99,999”) and 0 (“not”).
More than $100,000	Coded as 1 (“More than $100,000”) and 0 (“not”) [reference group].
*Race/ethnicity*	Measured by response to: “What is your race?”
Black	Coded as 1 (“Black”) and 0 (“not”).
Other	Coded as 1 (“American Indian; Alaska Native; Asian; Native Hawaiian or Other Pacific Islander; two more races” and 0 (“not”).
Hispanic	Coded as 1 (“Hispanic/Latino”) and 0 (“not”).
White	Coded as 1 (“White”) and 0 (“not”) [reference group].
*Educational attainment*	Measured by response to: “What is the highest degree or level of school have you completed?”
Less than HS diploma	Coded as 1 (“Less than HS diploma”) and 0 (“not”) [reference group].
HS diploma or GED	Coded as 1 (“HS diploma or GED”) and 0 (“not”) [reference group].
Some college or Associate's	Coded as 1 (“Some college or Associate's”) and 0 (“not”) [reference group].
Bachelor's	Coded as 1 (“Bachelor's”) and 0 (“not”) [reference group].
Master's, Professional, or Doctoral	Coded as 1 (“Master's, Professional, or Doctoral”) and 0 (“not”) [reference group].
*Household Composition*	
Number of children in household	Measured by response to: “How many members of your household are 0–17 years old?” Variable ranges from 0 to 4.
Number of elderly in household	Measured by response to: “How many members of your household are 65 years or older?” Variable ranges from 0 to 6.
Medical device	Measured by response to: “Are any medical devices used in your home, such as ventilators, nebulizers, or CPAP machines?” Respondents that reported “yes”, variable was coded as 1.
Not employed	Measured by response to: “Which best describes your current employment status?” Respondents that reported “not employed,” variable was coded as 1.
Retired	Measured by response to: “Which best describes your current employment status?” Respondents that reported “retired,” variable was coded as 1.
Female	Measured by response to: “What is your sex?” Respondents that reported “female,” variable was coded as 1.
No internet	Measured by response to: “In your home, do you or any member of your household have access to the Internet?” Respondents that reported “No access to the internet,” variable was coded as 1.
***Housing characteristics***	
*Housing tenure & type*	
Renter	Measured by response to: “Is your home …” Respondents that reported “Rented,” variable was coded as 1.
Homeowner	Measured by response to: “Is your home …” Respondents that reported “Owned by you or someone in your household,” variable was coded as 1 [reference group].
Single family home	Measured by response to: “Which best describes your home?” Respondents that reported “Single-family house detached from any other house” or “Single-family house attached to one or more other houses,” variable was coded as 1 [reference group].
Apartment	Measured by response to: “Which best describes your home?” Respondents that reported “Apartment in a building with 2–4 units” or “Apartment in a building with 5 or more units,” variable was coded as 1.
Mobile home	Measured by response to: “Which best describes your home?” Respondents that reported “Mobile home,” variable was coded as 1.
*Housing conditions*	
Poor insulation	Measured by response to: “Which of the following best describes the insulation level of your home?” Respondents that reported “Poorly insulated” or “Not insulated,” variable was coded as 1.
Adequate insulation	Measured by response to: “Which of the following best describes the insulation level of your home?” Respondents that reported “Adequately insulated” or “Well insulated,” variable was coded as 1 [reference group].
Drafty	Measured by response to: “How often do you or other members of your household find your home too drafty?” Respondents that reported “All the time,” “Most of the time,” or “Some of the time,” variable was coded as 1.
Built before 1980	Measured by response to: “About when was your home built?” Respondents that reported any category before 1980, variable was coded as 1.
Bult after 1980	Measured by response to: “About when was your home built?” Respondents that reported any 1980 and after, variable was coded as 1 [reference group].
Air conditioner	Measured by response to: “Is any air conditioning equipment used in your home?” Respondents that reported “Yes,” variable was coded as 1.
Space heater	Measured by response to: “Space heating equipment used?” Respondents that reported “Yes,” variable was coded as 1.
Electricity use, in kwh	Total electricity use, in kilowatt-hours, 2020, including self-generation of solar power
Electricity cost	Total electricity cost, in dollars, 2020
***Population density***	
Urban	2010 Census Urban Type Code Coded as 1 (“Urban cluster” or “Urban area”) and 0 (“not”).
Rural	2010 Census Urban Type Code Coded as 1 (“Rural area”) and 0 (“not”) [reference group].
***Climate***	
Heating degree days	Heating degree days in 2020, base temperature 65F; Derived from the weighted temperatures of nearby weather stations. Variable ranges from 0 to 17,383.
Cooling degree days	Cooling degree days in 2020, base temperature 65F; Derived from the weighted temperatures of nearby weather stations. Variable ranges from 0 to 5534.

## Footnotes

1 Each respondent has a state, division, and region identifier. Previous iterations of RECS did not allow for state-level estimates. To produce a nationally representative sample with state identifiers, the EIA provided base weights adjusted for survey ineligibility and nonresponse and additionally applied poststratification adjustments to ensure RECS weights matched U.S. Census Bureau state-level estimates [79].

2 There were too few observations to complete this analysis with required medical assistance as the outcome measure.

3 As a sensitivity check, I add receipt of energy assistance in 2020. Results from 2019, 2018, 2017, and 2016 remain the same; however, having broken equipment in 2020, receipt of a disconnection notice in 2020, and being disconnected from the utility provider in 2020 were all positively associated with receipt of assistance in 2020.
